# Peripheral Osteoma: A Report of a Case in the Anterior Mandibular Region

**DOI:** 10.7759/cureus.48310

**Published:** 2023-11-05

**Authors:** Carlo Maksoud, Elissa Akouri, Chirine Chammas, Georges Aoun

**Affiliations:** 1 Oral Medicine and Maxillofacial Radiology, Lebanese University, Beirut, LBN; 2 General Dentistry, Lebanese University, Beirut, LBN

**Keywords:** mandible, tumor, benign, bone, peripheral osteoma

## Abstract

Osteomas are benign lesions that arise from the proliferation of either cancellous or compact bone. They can be categorized as extra-skeletal, peripheral, or central. Extra-skeletal soft tissue osteomas often develop within muscles, while peripheral and central osteomas emerge from the periosteum and endosteum, respectively. Osteomas are usually asymptomatic and rarely affect the jaws. In the mandible, they typically occur in the posterior regions. In this report, we present the case of a 46-year-old female with a mandibular peripheral osteoma located in the anterior region and treated by total resection via median cervicotomy.

## Introduction

Osteoma is an osteogenic tumor that arises from bone proliferation. It can develop in any bone but rarely affects facial bones. It is benign and has slow and generally limited expansive growth [1,2]. This tumor is the prevailing noncancerous growth found in the nasal cavity and paranasal sinuses, and it is also the most frequent type of tumor in the frontal sinus [3].

Despite the fact that this lesion is usually asymptomatic, it can cause speaking and eating impairments, headaches, neuralgia, exophthalmos, diplopia, sinusitis, epistaxis, pain, swelling, and asymmetry, depending on its location and size [1,4]. Otherwise, the discovery of the lesion is incidental during clinical or radiological examination [5].

Osteomas can be classified as central, peripheral, or extra-skeletal. Central osteomas originate from the endosteum, whereas peripheral osteomas develop from the periosteum, and extra-skeletal soft tissue osteomas typically form within muscles [6].

Given the fact that osteomas are radiopaque lesions, the differential diagnosis includes exostoses, peripheral ossifying fibroma, periosteal osteoblastoma, osteoid osteoma, periosteal osteosarcoma, focal sclerosing osteomyelitis, osteochondromas, osteophytes, and condylar hyperplasia [1,5,7].

There are no documented cases of malignant transformation in osteomas. If treatment is recommended, surgery is usually the chosen course, and recurrence is uncommon [8].

## Case presentation

A 46-year-old patient was referred by her stomatologist to the oral and maxillofacial surgery department, complaining of a hard, painless, slowly growing swelling in the submental region that had been present for approximately one year. The swelling had slowly increased in size to attain his actual size. It was not associated with paresthesia, fever, or difficulty speaking or eating. There was no history of infection or trauma; there were no other swellings present in any other part of the body; and the patient's medical background did not provide any relevant information.

On physical examination, a single well-defined nodule of approximately 3 cm in diameter could be palpated behind the chin. It was obvious that the nodule was closely connected to the basal border of the mandible. The skin covering the tumor as well as the intraoral and cervical examinations were normal.

**Figure 1:**
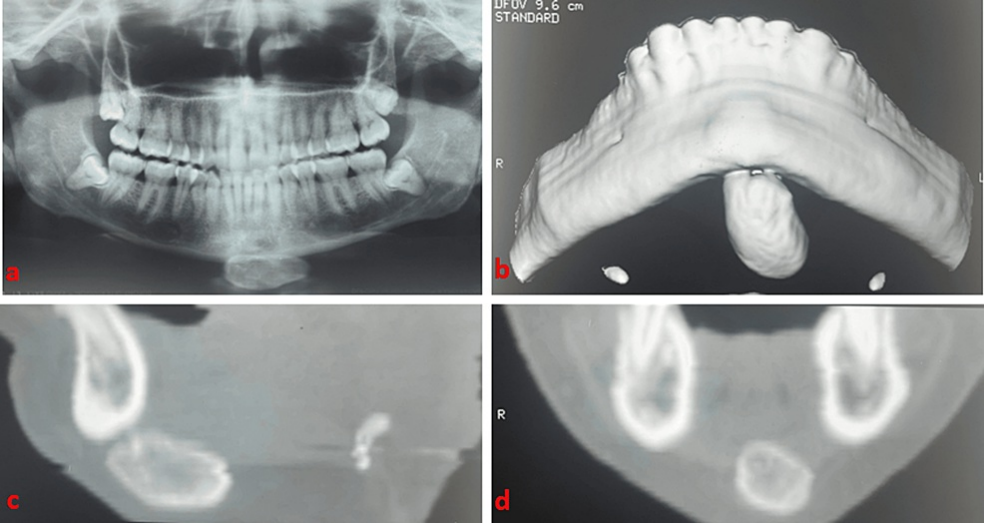
(a) Panoramic radiograph; (b) 3D CT image; (c) sagittal CT image; and (d) coronal CT image.

The performed radiographs demonstrated a radiopaque, well-circumscribed, oval mass (antero-posterior axis) with distinct borders, pedunculated, in contact with the inferior mandibular border (Figure 1).

A total resection via median cervicotomy was performed. Since the lesion was extending antero-posteriorly, lying in the region of the anterior bellies of the digastric muscle, the surgery was done with extra-oral access under general anesthesia. An incision of about 3 cm was performed in the submental area. It should be mentioned that the nasotracheal intubation was hard due to the extent of the mass in the epiglottis.

**Figure 2:**
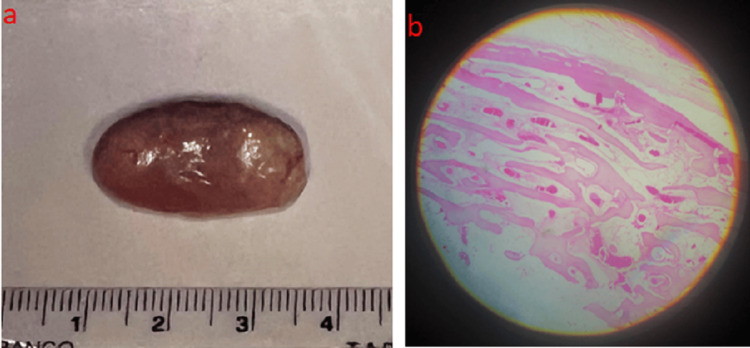
(a) Macroscopic specimen; (b) histopathologic image showing Haversian canals of mature bone with fat compartments (hematoxylin-eosin stain ×40).

The specimen was sent for histopathological examination, which revealed canals of mature bone with fat compartments, supporting the diagnosis of peripheral osteoma (Figure 2).

The conclusive histopathological examination of the specimen confirmed the presence of an osteoma.

## Discussion

Osteoma is a benign tumor made up of well-differentiated cancellous or compact bone [9]. Osteomas can exhibit areas resembling osteoblastomas, making it difficult to differentiate them from true osteoblastomas. Some experts suggest that osteomas with osteoblastoma-like characteristics might display more aggressive behavior. The majority of osteomas are small in size, although in exceptional instances, they can grow to a significant extent, leading to displacement and potential harm to nearby structures. Lesions with a diameter exceeding 3 cm are considered giant lesions [1].

Osteomas are most commonly found in the frontal, ethmoid, and maxillary sinuses [10]. Concerning the jawbones, the mandible is more frequently affected than the maxilla [3]. Mandibular cases typically occur in the angle region and condyle, followed by the ramus and the molar-premolar area [2].

While osteomas can manifest at any age, they are predominantly observed in young adults, and a notable thing about osteomas is their capacity to continue their growth even during adulthood, unlike other bony exostoses [1].

In cases of multiple osteomas of the jaws, Gardner’s syndrome, which is an autosomal dominant disease, should be suspected [11]. The patient usually experiences manifestations such as abdominal pain, rectal bleeding, and diarrhea. The presence of colorectal polyposis, skeletal anomalies, and supernumerary or impacted teeth constitutes the characteristic triad for this syndrome [7]. The syndrome typically emerges during the teenage years, and there is a high likelihood of malignant transformation of colorectal polyps by the age of 40 [4]. In certain cases, early detection of the syndrome can be a life-saving event since osteomas often appear before colorectal polyposis. Mandibular osteomas could serve as a genetic indicator for the onset of colorectal carcinoma [1].

Concerning peripheral osteoma, it is a rare condition that primarily manifests in young individuals, with no sex predilection [4]. It is the most common type encountered in the jaw [12]. This lesion is usually unilateral and pedunculated, giving it a mushroom-like aspect [1]. The etiology is still unclear, but three hypothetical theories have been proposed. The first one suggests that this lesion is developmental, meaning that congenital anomalies are responsible for its occurrence. However, considering that the majority of reported cases are adults, this theory appears improbable, as genetic conditions typically manifest during the developmental stage of growth. The second hypothesis, which is also no longer accepted, posits that an osteoma is a neoplasm resulting from chronic inflammation. However, if this were true, all osteomas would exhibit rapid and unrestricted growth, which is not the case. The third suggestion, namely the reactive theory, which combines trauma and muscle activity, has been widely accepted as the most plausible explanation for the development of peripheral osteoma. According to this theory, trauma results in subperiosteal bleeding, while muscle traction locally elevates the periosteum, triggering an osteogenic response [13]. The masseter is usually the muscle involved [4]. The initiating trauma is often forgotten by the patient because it is usually minor. However, the ongoing muscle traction in the affected area could sustain the reaction, leading to tumor growth [14]. That’s the reason why peripheral osteomas are mostly found in the lower border or buccal region of the mandible, which are areas prone to trauma. However, while trauma, inflammatory, endocrine, and infectious processes are frequently mentioned as potential causes in the literature, no specific etiological factor can be linked to this particular case [1,4].

## Conclusions

Osteomas are slow-growing, asymptomatic, benign tumors rarely involving the craniofacial region. They infrequently affect the jaws, and mandibular cases usually occur in the posterior regions. Surgery is only indicated if they produce facial asymmetry or discomfort. Recurrence is rare; however, radiographic follow-up at a six-month interval is recommended for at least two years, in addition to two annual radiographs thereafter. In the present case, we reported the case of an osteoma of the anterior mandibular region.
